# Hyperhomocysteinemia and the role of B vitamins in cancer

**DOI:** 10.2478/v10019-010-0022-z

**Published:** 2010-05-24

**Authors:** Nadja Plazar, Mihaela Jurdana

**Affiliations:** College of Health Care Izola, University of Primorska, Izola, Slovenia

**Keywords:** homocysteine, hyperhomocysteinemia, B vitamins, cancer

## Abstract

**Background:**

Patients suffering from malignancies have increased complications due to corresponding cardiovascular diseases and risk factor for the development of venous thromboembolism. Epidemiological studies have shown that increased homocysteine plasma concentration (hyperhomocysteinemia) is related to a higher risk of coronary heart disease, stroke, peripheral vascular disease and malignancies. Homocysteine (tHcy) is an intermediate sulfur-containing amino acid produced from methionine during processing of dietary proteins. The plasma homocysteine levels are strongly influenced by diet, as well as by genetic factors. Folic acid, vitamins B6 and B12 are dietary components which influence the plasma homocysteine levels the most. Several studies have found that high blood levels of B vitamins are related to the integrity and function of DNA, and, are at least related to lower concentration of homocysteine. Folate depletion has been found to change DNA methylation and DNA synthesis in both animal and human studies. Because of this critical role of folate, most studies including homocysteine have focused on these two actions.

**Conclusions:**

Hyperhomocysteinemia proves to be the most common condition highly associated with both venous and arterial thrombosis in many cancer patients, while the associated pathophysiology has not been precisely established yet. Therefore, of current interest is the possible role of folate metabolism developing into a cancer initiating hyperhomocysteinemia. This review will discuss this possibility.

## Introduction

It has been long postulated that the plasma homocysteine concentration is inversely related to the occurrence of cardiovascular and cerebrovascular diseases.^1^ More recently, increased plasma homocysteine concentration has been postulated as a risk factor for cancer and even as a novel tumour marker.^2^ This increased risk can be attributed to the high prevalence of classical factors in these patients, such as hypertension, diabetes, and dyslipidemia, but most certainly (also) to factors resulting from the malignant disease and the applied selected therapy. For example, back in 1865 Trousseau described hypercoagulability and increasing risk of »spontaneous coagulation« in patients with cancer.^3^ Nowadays, it is established that breast, pancreas, and gastrointestinal cancers are associated with a higher incidence of thrombosis. With more advanced stages of cancer there is a lower overall survival rate^4^, but, also a greater risk of venous thromboembolism^5^, what can additionally influences on the survival of patients. A raised plasma homocysteine level is associated with serum B vitamins concentration, especially folate levels, since these are required in homocysteine metabolism. Adequate B vitamins intake is essential for nucleotide biosynthesis, DNA replication and methyl group supply and thus for cell growth and repair.^6^ Evidence suggests that folate depletion fosters the development of cancer, particularly colorectal cance^6^–^10^, whereas high doses of folic acid may enhance growth of cancer cells.^7^ However, the complexity of the folate metabolic pathway may suggest that different metabolites of folate might induce multiple effects in normal, preneoplastic and malignant cells.

## Homocysteine, metabolism and cardiovascular complications

**FIGURE 1:**
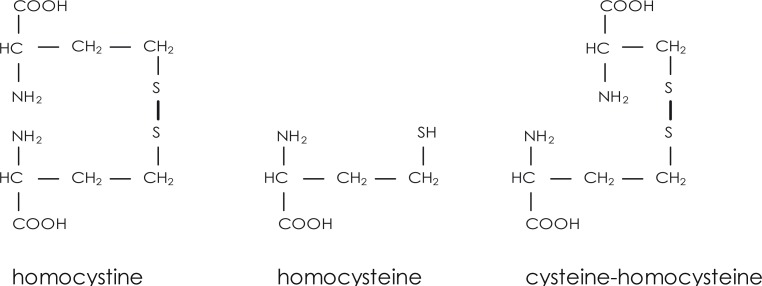
Structural formulae: Homocysteine, homocystine and mixed disulfide (cysteine-homocysteine).

Proliferating cells secrete more homocysteine compared to non-proliferating cells. Homecysteine is a sulphur-containing intermediate in the normal metabolism of the essential amino acid methionine present in almost all body cells and mostly 5 to 10% of daily synthesized homocysteine (1.2 mmol/day)^11^ is transferred into the blood through hepatocytes. The thyol group of homocysteine makes it readily available to be oxidized in the blood at physiological pH upon which it forms disulfide bonds with other thyols (Figure 1).^12^

In a healthy population the frequency of moderate hyperhomocysteinemia (12–30 μmol/L) is 5 to 7%, with higher values for men being attributed to gender differences like estrogen presence in women. This is confirmed by the fact that after menopause the blood levels of homocysteine of woman approximate those in men.^13^ Another cause for moderate hyperhomocysteinemia is an unbalanced diet with suboptimal intake of vitamins (B6, B12 and folates), acting as coenzymes in the metabolism of homocysteine.^14^,^15^ In the elderly, such a moderate hyperhomocysteinemia due to lack of vitamins B and folates is very common. A survey, carried out by Herrmann *et al*., showed that 32% of healthy elderly people aged 65 to 75, and 58% of those over 85 years of age suffer from hyperhomocysteinemia, indicating that hyperhomocysteinemia significantly increases with age^16^, therefore it decreases in younger people as the incidence of malignancies.^17^

**FIGURE 2:**
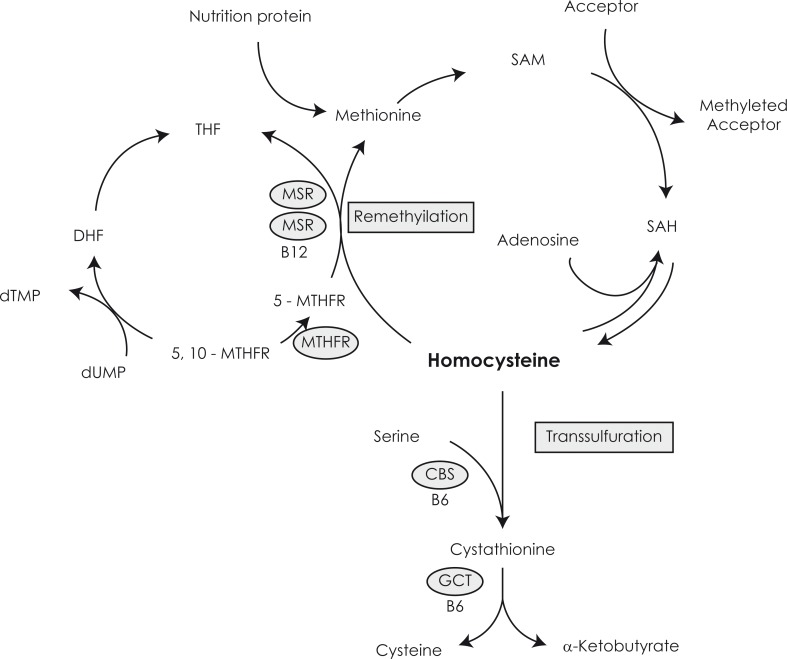
Metaboliolism of homocysteine. dUMP - desoxyuridine monophosphate, dTMP - desoxytimidine monophosphste, THF - tetrahydrofolate, DHF - dihydrofolate, 5-MTHF - 5-methyltetrahydrofolate, 5,10-MTHF - 5,10-methyltetrahydrofolate, 5,10 MTHFR - 5,10- methyltetrahydrofolate reductase, MS - metionin synthase, MSR - metionin synthase reductase, B12 - vitamin B12, SAM - S-adenosylmethionine, SAH - S-adenosylhomocysteine, CBS - cystathionine β-synthase, GCT - γ-cystathionase, B6 - vitamin B6.

Via the trans-sulfuration pathway homocysteine is converted into cystathionine to form cysteine by cystathionine-ß-synthase, with vitamin B6 as a co-factor. Another pathway of homocysteine metabolism is the re-methylation pathway, which is connected with the folate metabolic pathway. It involves the transfer of a methyl group from 5-methyl-tetrahydrofolate to homocysteine to form methionine, and eventually S-adenosylmethionine. The methyl transfer from 5-methyl-tetrahydrofolate to homocysteine is catalyzed by methionine-synthase, and requires vitamin B12 as a cofactor (Figure 2). Important to notice is that S-adenosylmethionine is the universal methyl donor for methylation reactions. The resulting tetrahydrofolate transfers into the 5.10 methyltetrahydrofolate with the enzyme 5.10 methyltetrahydrofolate reductase (MTHFR) and then into the 5 methyltetrahydrofolate (5-MTHF).^18^ Cellular availability of 5-MTHF may be of great importance in regulating cellular effects of homocysteine related to cell growth. Therefore, deficiencies of folate and vitamin B12 and reduced activity of the involved metabolic enzymes will inhibit the breakdown of homocysteine, which will lead to an increase of the intracellular homocysteine concentration.^19^

Moreover, hereditary causes of increased homocysteine blood concentrations exist (hyperhomocysteinemia). Most studies refer to changes in the genes for those enzymes that lead to severe hyperhomocysteinemia, such as the CBS gene (cistation-β syntases) or in the GCT gene (γ cistationase), both coding the trans-sulfuration pathway. Further, mutations do occur in genes coding for enzymes involved in the remethylation pathway and the related folate metabolic pathway. For a homozygous person with a mutation MTHFR 677C => T the enzyme activity is reduced to 35% of the normal.^20^ A typical mutation in Europe occurs within the gene for MTHFR 677C => T, with different incidences between German (24.5%) and Italian (43.8%) populations.^20^ Moreover, it seems that this mutation and the reduced activity of the enzyme MTHFR are not connected with hyperhomocysteinemia if persons have balanced diet with optimal intake of vitamins (B6, B12 and folates).^19^

Hyperhomocysteinemia is frequently associated with folate deficiency and it has been long postulated that the plasma homocysteine concentration is inversely related to the occurrence of cardiovascular disease and venous thrombosis.^21^–^24^

## Disturbed homocysteine metabolism, hyperhomocysteinemia and cancer

Hyperhomocysteinemia is commonly occurring in a wide range of unrelated diseases. For example, in patients with renal failure a strong, positive correlation was observed between homocysteine (tHcy) levels, serum creatinine, and the renal glomerular filtration rate. Rheumatoid arthritis impaired gastric and other disturbances results often in elevated blood tHcy.^25^ The disease among which elevated tHcy are observed are: Systemic lupus erythematosus, non-insulin-dependent and insulin-dependent diabetes mellitus, hypothyroidism, cognitive impairment and neuropsychiatric disorders (dementia, depression, schizophrenia), fibromyalgia and chronic fatigue syndrome, Parkinson’s disease, cerebrovascular disorders, and aseptic meningitis.^26^

Increased tHcy levels are often found in patients with neoplastic diseases.^27^ *In vitro* it was shown that some cancer cell lines are incapable of remethylating tHcy and it was recently shown that ovarian cancer cells from patients with elevated tHcy have impaired capacity to remethylate tHcy.^28^,^29^ Tempting to conclude that hyperhomocysteinemia in cancer patients could be secondary to the cancer. However, impaired methylation of DNA and polyamines has often been proposed to be involved in carcinogenesis^30^, so the combination of increased tHcy levels and impaired methylation capacity in patients has been proposed as being carcinogenic.^31^ In lymphocytes, positive correlation between cellular tHcy levels and increased chromosome damage was shown.^31^

Patient with malignancies often have an increased risk of venous thromboembolic (VTE) disease^5^ and as such being the second most common cause of death in cancer patients, second to the primary disease itself. The pathophysiology of this association has not been precisely defined. However, it has been reported that in cancer patients several pro-coagulant factors are increased.^32^ Other established contributors to the VTE increased risk is oncological therapy as chemotherapy, hormonal adjuvant therapy, surgery, central venous catheters, immobility and inherited thrombophilia.^5^,^33^,^34^ However, this oncological therapy can also influence the immunological response of treatment patients.^35^ In women with advanced breast cancer hyperhomocysteinemia is common.^36^ This observation could explain the high rate of venous thrombosis in women with metastatic breast malignancy.^6^ Furthermore, the association between MTHFR C677T polymorfism and breast cancer has been reported. However a positive correlation has not been confirmed by all studies.^37^ MTHFR C677T polymorphism is associated with changes in intra-cellular folate cofactors, affecting DNA methylation and synthesis via altered one-carbon transfer reactions. Of further notice on this association are potential ethnic differences.^38^

Al-Awadi *et al*.^39^ demonstrated with nude mice implanting human breast, prostate and pancreas tumour cells leads to decreased plasma cysteine, homocysteine and methionine levels over a two-month period, which was a direct result from the progressing implanted tumour cells. In the case of methionine, the decrease was significant only due to progression of the breast tumours over a long time period. The results suggest that the sulphur amino acids cysteine, homocysteine and methionine can be potentially used as plasma or serum biomarkers for cancer progression.

Many other studies showed that the raised tHcy is related to the cancer itself and to the extent of the disease.^21^,^40^ After remission of the cancer in children with acute lymphoblastic leukemia the tHcy levels returned to normal.^2^

Both plasma concentration of homocysteine and neopterin, a catabolic product of guanosine triphosphate-GTP and as such an immune system activation marker, are closely associated and elevated in patients with various types of disease.^3^ From *in vitro* studies it has been shown that tumour cells and other proliferating cells release homocysteine.^,^^41^ This *in vitro* notion might be extrapolated to the *in vivo* situation and could explain why hyperhomocysteinemia is observed in patients with various kinds of cancers. Within cellular immune activation, T cells release large amounts of the cytokine IFN-γ, which stimulates human monocyte-derived macrophages and dendritic cells to produce neopterin.^42^ For example, in cancer patients, increased urine and plasma neopterin concentrations have been reported, suggesting enhanced cellular immune activation.^43^,^44^ Therefore, immune activation cascades might also be an important triggers for the accumulation of plasma homocysteine in various diseases, including malignancies.

When different tumours are compared, the frequency of increased neopterin concentration is much lower in patients with breast cancer that it is in patients with other types of cancers.^3^ Although no association between neopterin and tumour size or lymph node status has been shown in women with breast cancer, follow-up examinations reveal that at diagnosis high urine neopterin concentrations are associated with shorter survival.^4^ However, these authors conclude that plasma homocysteine and neopterin concentrations are only rarely elevated in breast cancer patients and they note that the activation and proliferation of immunocompetent cells rather than tumour cells proliferation is responsible for hyperhomocysteinemia in these breast cancer patients.

## B vitamins for cancer prevention

Folate, vitamin B12, and vitamin B6, have a number of biologic roles that make them potentially important in cancer. Within DNA synthesis they function as coenzymes in the synthesis of purines and thymidylate. Diminished levels of these vitamins may result in misincorporation of uracil into DNA, leading to chromosome breaks and disruption of DNA repair.^45^ As explained earlier both folate and vitamin B12 are involved in DNA methylation. Deficient folate and vitamin B12 levels can reduce the availability of S-adenosylmethionine, the universal methyl donor, for DNA methylation and may thereby influence gene expression.

Inadequate body levels of biologically active folate, vitamin B6, and vitamin B12 are primary determinants of high blood homocysteine levels.^46^ Folic acid is a component of food which has been associated with lower cancer risk in epidemiologic studies.^9^,^10^,^47^,^48^ Wide geographical variation and migrant studies in cancer incidence and mortality suggest that diet and other lifestyle factors as physical activity influence cancer risk.^49^,^50^ Data on cancer incidence and mortality are available from 37 countries and analysis showed that incidence of colorectal cancer was inversely correlated with more cereals (grains) in the diet.^51^ Folic acid present in a wide variety of plant foods, such the legumes, vegetables, fruits and whole grains is thought to be protective against colorectal cancer.^52^,^53^ The lack of folic acid in animal cells studies resulted in DNA defects that resemble effects found in cancer cells.

It has been hypothesized in many epidemiologic studies^38^,^54^,^55^ that cancer can be initiated by DNA damage (increasing DNA methylation, and by repairing and reducing formation of DNA strand breaks of p53 and Apc genes) caused by folic acid deficiency.^56^,^57^ A deficiency of folic acid leads to a low level of thymidilic acid and alterations in the pool of nucleotides available for DNA and RNA synthesis. It is even suggested that adequate folate intake may be important in the prevention of breast cancer, particularly among women who consume alcohol.^58^–^60^ Alcohol is a known folate antagonist and thus could increase an individual’s requirement for folate intake. For vitamin B12, unlike folate, variation in amount absorbed rather than intake is the main determinant of plasma levels in Western populations.^61^ In a prospective study analysis of collected blood from 195 case–control pairs, low plasma levels of vitamin B12 were associated with increased risk of breast cancer among post-menopausal women; however, low plasma levels of folate, and homocysteine were not associated with breast cancer risk.^62^ Hypofolatemia and metabolic alteration in homocysteine, vitamin B12 could be associated with laryngeal cancer.^63^ Therefore, a great effort was made to proof this association as it was made to find association between cysteine cathepsins as well as stefins and promoting and invasion of head and neck tumours.^63^,^64^ Increased plasma vitamin B12 concentration may reduce the risk of rectal cancer.^65^

A recent animal study demonstrated that a B12-deficient diet, which was of insufficient severity to cause anemia or illness, disturbed normal homeostasis of one-carbon metabolism in the colonic mucosa and resulted in diminished genomic DNA methylation and increased uracil misincorporation in DNA, both of which are purported mechanisms for one-carbon metabolism-related colonic carcinogenesis.^66^

In a large prospective study on health care professionals, high intake of folic acid was found to be significantly correlated with low incidence of colorectal adenomas (polyps).^67^ Therefore the diet regime and life style factors should be consider as primary prevention beside also important secondary prevention.^49^,^50^,^68^ Case control studies have as well as found high folic acid intake to be correlated with low risk for either pancreatic cancer or breast cancer.^69^,^70^ In Greece and in Argentina studies correlating breast cancer and diet found risk reductions from six to ten fold in subjects eating mostly vegetables rich of B vitamins.^71^ The high risk of developing cancer in a lifetime in the North American and Western European societies might be related to the low intake of vegetables and particularly folic acid might be lacking in diets. Folic acid deficiency could be the permissive condition that enables DNA damage to occur and accumulate. This can lead to DNA damage and cancer.

## Is synthetic folate fortification always good for us?

Many countries have implemented mandatory folic acid fortification of flour and grain products to reduce the risk of various diseases, especially neural-tube birth effects. Experimental evidence suggests that high doses of folic acid may enhance growth of cancer cells.^7^,^53^,^72^,^73^ These effects have resulted in substantial increase in circulating folate and unmetabolized folic acid concentration.^72^,^74^ Described the beneficial effects of folate in preventing cancer, it is also well known that high intake of synthetic folic acid might mask vitamin B12 deficiency.^74^ Experimental studies suggest that excessing folic acid may promote the progression of already existing preneoplasms.^7^ Responsible mechanisms of high folates concentration causing cancer promoting effects include folates providing nucleotide precursors for the preneoplastic cells improving their replication and proliferation. Folates, methyl donors might lead to a de novo methylation and subsequent inactivation of tumour-suppressor genes, resulting in accelerated tumour progression.^7^ The safe upper limit for folate intake as well as the safe upper folate concentration in blood are not known.^6^ The mandatory fortification of food with folic acid, it dose and the time of intervention depends by the country’s decision.^72^

Because the safety of folate might depend on its chemical structure (natural folate or synthetic folic acid), there is the question of potential adverse effects of circulating unmetabolized folic acid.^75^

## Conclusions

Many data support a relationship between hyperhomocysteinemia, low B vitamins concentration and risk for various types of cancer. Defective metabolism of tHcy in carcinogenesis is well documented, but the pathophysiology of this association is not fully understood. Many authors suggest that factors contributing to folate status are not protective against certain type of cancer, so further studies are needed to explore folate studies in human.
